# A community partitioning algorithm for cyberspace

**DOI:** 10.1038/s41598-023-46556-4

**Published:** 2023-11-03

**Authors:** Kai Qi, Heng Zhang, Yang Zhou, Yifan Liu, Qingxiang Li

**Affiliations:** Institute of Geospatial Information, PLA Strategic Support Force Information Engineering University, Zhengzhou, 450001 Henan China

**Keywords:** Computer science, Information technology

## Abstract

Community partitioning is an effective technique for cyberspace mapping. However, existing community partitioning algorithm only uses the topological structure of the network to divide the community and disregards factors such as real hierarchy, overlap, and directionality of information transmission between communities in cyberspace. Consequently, the traditional community division algorithm is not suitable for dividing cyberspace resources effectively. Based on cyberspace community structure characteristics, this study introduces an algorithm that combines an improved local fitness maximization (LFM) algorithm with the PageRank (PR) algorithm for community partitioning on cyberspace resources, called PR-LFM. First, seed nodes are determined using degree centrality, followed by local community expansion. Nodes belonging to multiple communities undergo further partitioning so that they are retained in the community where they are most important, thus preserving the community’s original structure. The experimental data demonstrate good results in the resource division of cyberspace.

## Introduction

As cyberspace continues to develop, the amount of data about various internet resources is also increasing. To better manage, maintain, and optimize cyberspace, it is necessary to conduct an in-depth analysis of these resources and explore their internal connections. If large-scale cyberspace resource data is analyzed using traditional text, numbers, and charts, it is difficult to extract valuable resource information. To better perceive and describe cyberspace resource information, it is divided into communities, and the huge and complex network topology structure is divided into several relatively independent parts. This approach facilitates the management, maintenance, and optimization of the network. This division of resource communities can help us understand the hierarchical structure of the network and determine the important nodes and key paths in the network, allowing us to better optimize network performance, improve network security, and facilitate the location and processing of network faults^1–4^.

Community partitioning methods may be divided into non-overlapping and overlapping community detection methods, depending on their scope of application. A non-overlapping community only contains nodes belonging exclusively to the community, whereas an overlapping community contains nodes that are simultaneously members of two or more communities^5^. Non-overlapping community detection algorithms mainly include: modularity optimization algorithms^6–8^, spectral analysis algorithms^9–11^, information theory algorithms^12–14^, and label propagation algorithms^15–17^. When a non-overlapping community detection algorithm assigns a node to a specific community, the node would not be considered for inclusion in other communities. However, real-world cyberspace community structures often have numerous nodes participating in two or more communities. As a result, overlapping community detection algorithms more accurately reflect the community structure of cyberspace^18–22^. Furthermore, overlapping nodes are often more important for various communities than non-overlapping nodes, which implies that their selection and management can influence the overall quality of the community partition. Overlapping community detection algorithms may be classified as global^18,23,24^ or local information-based community detection algorithms^25–27^. The global information-based algorithms, while effective, require traversal of the entire network in each iteration. This results in high time complexity and renders them unsuitable for real large-scale networks. To reduce the time complexity of community detection, Lancichinetti et al. proposed the local information-based local fitness maximization (LFM) algorithm^26^, which introduces a fitness function and a community-size parameter, enabling the detection of overlapping community structures at different levels. Although LFM is very fast and can analyze large-scale networks with millions of nodes, it randomly selects seed nodes for local community expansion during community partitioning. Consequently, it produces a different community partitioning in every run. Due to the differences between network structures and properties, no unique community detection algorithm can be universally adapted to various networks with high accuracy^28^. Therefore, numerous scholars have carried out a series of studies on different network and purpose needs. Berahmand et al. proposed the Augment Graph Regularization Nonnegative Matrix Factorization for Attributed Networks (AGNMF-AN) community partitioning algorithm^29^. Ma et al. proposed a multi-layer community discovery algorithm based on joint non-negative matrix decomposition^30^. Zhu et al. proposed a modularity optimization algorithm based on k-plex, which can accurately identify small community structures^31^. Zhe et al. used a greedy maximization modularity algorithm to partition the community of network topology and attribute information^32^. Bahulkar et al. found the community structure by optimizing the local modularity of the community^33^. Zhang et al.'s agglomerative approach used the concept of real connections to discover overlapping communities in the network^34^.

The common methods used for identifying key nodes in a community include degree centrality^35^, betweenness centrality^36^, closeness centrality^37^, and eigenvector centrality^38^. However, none of these measures account for the directionality of node-to-node information transfers in the cyberspace community structure. Google introduced the eigenvector centrality-based PageRank (PR) algorithm^39^, which utilizes the concept of random walks in graph theory^12^. PR views the network as a directed graph, with each webpage being a node and each link being a directed edge, and ranks the webpages according to their importance, which is determined from their linkage relations with other webpages.

The traditional community division algorithm only considers the structure of the network and does not consider the attributes of the community structure in the cyberspace. If the traditional community division algorithm is used, it cannot accurately divide the resources in the cyberspace. In a real cyberspace community structure, there are often numerous nodes that exist in two or more communities, and the transmission of information in cyberspace is characterized by direction. Therefore, we propose a community partitioning algorithm of network resource mapping, PR-LFM (Pagerank-local Fitness Maximization), which integrates the improved LFM algorithm with PR algorithms. Among them, the LFM algorithm can quickly discover communities based on local information and analyze large-scale networks with up to one million nodes. To better mine the association between nodes and communities, this method simultaneously considers the degree of connection between nodes and the similarity between nodes and communities. The PageRank algorithm not only considers the degree value of nodes, but also the contribution degree of nodes, and it can calculate the ranking of nodes according to the connection relationship of nodes in the network. Furthermore, it exhibits robust performance in the face of large-scale and complex networks. In this study, the PR-LFM algorithm first selects the seed node as the initial community based on the degree central value and then expands the initial community according to the fitness function. Subsequently, by comparing the importance degree of the overlapping community where the overlapping node resides, the overlapping node is retained in the more important community, thereby achieving the same effect as the traditional non-overlapping community division algorithm. Experiments demonstrate that, compared with the traditional non-overlapping community partitioning algorithm, the proposed algorithm takes into account the hierarchy, overlap, and direction of information transmission in the cyberspace and preserves the original community structure in the cyberspace better.

## Methods

### Cyberspace community structure

Cyberspace is a virtual domain distinct from traditional geographic space, as it is an information network devoid of concepts of distance. Information within it spreads along specific paths and directions, with all kinds of events and processes occurring instantaneously and at zero distance. Hence, cyberspace is virtual, dynamic, directional, and open^40^. The main focus of cyberspace mapping is the community structure of its nodes, which provide insight into the internal workings of cyberspace.

**Figure 1:**
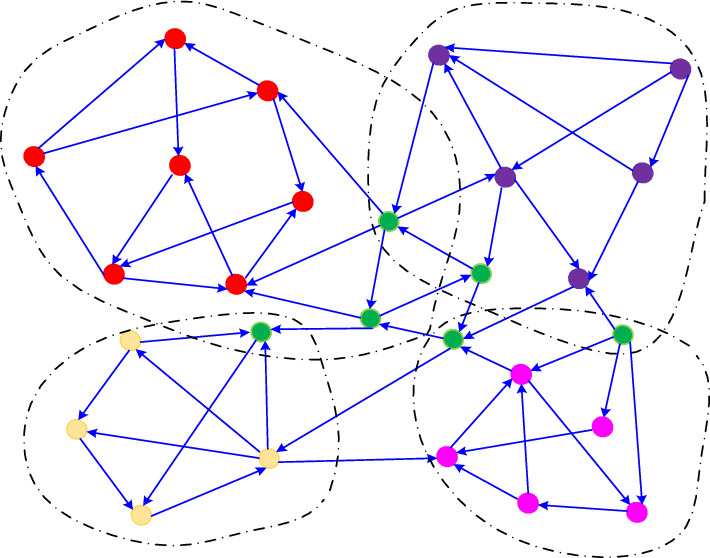
Overlapping of cyberspace communities. Each color represents a different community, and the directed edges represent the direction of information transfer between a pair of nodes. Green nodes are overlapping nodes that are members of two or more communities.

The community structure of cyberspace is characterized by hierarchy, overlaps^41^, and directionality. In this context, hierarchy refers to nodes in the network having different levels of organization or structure. For instance, larger communities may contain smaller communities, which could in turn contain even smaller communities^42^. “Overlaps” in the community structure of cyberspace arise from the intersections that exist between different communities in their members, goals, and activities. “Directionality” refers to the fact that information flows from one cyberspace node to another are generally directional. Figure 1 illustrates four overlapping communities. Here, each color represents a different community, and the directed edges represent the direction of information transfer between a pair of nodes. Green nodes are overlapping nodes that are members of two or more communities.

### Importance metrics for directed nodes in cyberspace

As information flows in cyberspace are generally directional, we borrowed an idea from Google’s PR algorithm, i.e., webpages linked to many high-quality webpages must also be high-quality webpages. In other words, cyberspace nodes shall be evaluated based on the idea wherein nodes linked to important nodes must also be important nodes themselves.

#### Random walk model for cyberspace

The random walk model is a Markov process with states that randomly move at discrete time intervals. The probability of a state being chosen at each interval is determined by the state transition matrix *M*. If all nodes connected by a directed edge to a node have equal transition probabilities^12^, M is then a matrix of order *n*:

1 $$\begin{array}{*{20}c} {M = \left[ {m_{ij} } \right]_{n \times n} .} \\ \end{array}$$

**Figure 2:**
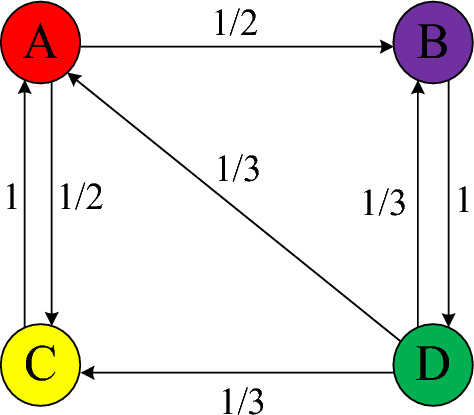
Random walk model for cyberspace. Nodes A, B, C, and D represent different nodes in cyberspace. The directed edges between the nodes represent the direction of linkage in cyberspace, while the weight of each edge represents the probability of random transition between its pair of nodes. For example, if some information is transferred to Node A, its transition probabilities to Nodes B and C are both 1/2. If the information is transferred to Node B, its transition probability to Node D is 1. From Node C, the transition probability to Node A is 1; from Node D, there is a 1/3 probability of transition to one of Nodes A, B, and C.

The value of ${m}_{ij}$ depends on the directed edges between the nodes. If the node *j* has *k* outdegrees and the node *i* is one of its sinks (*i*, *j* = 1, 2,…, *n*), the value of *m*_*ij*_ is *1/k*; otherwise *m*_*ij*_ is 0. A random walk model in cyberspace is shown in Fig. 2.

One may thus obtain *M*, Eq. (2), where each column represents the out node of a node, that is, the links of the current node to other nodes, which sum to 1. Conversely, each row represents the in node of a node, that is, the link from other nodes to the current node.

2 $$\begin{array}{c}M=\left[\begin{array}{cccc}0& 0& 1& 0\\ 1/2& 0& 0& 1/3\\ 1/2& 0& 0& 1/3\\ 0& 1& 0& 1/3\end{array}\right]\end{array}$$

The probability of the random walk process visiting some node at time *t* is given by the state distribution of its Markov chain at time *t*, which can be represented by an *n*-dimensional column vector ${R}_{t}$. Thus, the probability distribution ${R}_{t+1}$ for each node being visited at time *t* + *1* satisfies

3 $$\begin{array}{c}{R}_{t+1}=M{R}_{t}.\end{array}$$

#### Measurement of node importance in cyberspace using the PR algorithm

The PR algorithm depicts node linkages as a directed graph *A* = *(V,E)*, where *V* and* E* denote the vertices and directed edges of cyberspace, respectively. A random walk model, or a first-order Markov chain, is introduced to represent the process of information transfer between cyberspace nodes. This model can be used to evaluate node importance in cyberspace. The transition matrix of a general random walk model comprises a linear combination of two parts: one is the basic transition matrix *M* of the directed graph, which assigns equal transfer probabilities from one node to all its sinks, and the other is the random transfer matrix, which has a transition probability of *1/n* from one node to any other node. This general random walk Markov chain has a stationary distribution, denoted as *R*. The stationary distribution vector *R* is defined as the general PR of this directed graph^39^.

4 $$\begin{array}{c}R=d.M.R+\frac{1-d}{n}.1\end{array}$$

The first term in Eq. (4) represents the probability of each node being visited according to the transfer matrix M, and the second term denotes the completely random probability of each node being visited. *d* is the linear combination coefficient, and when *d* is close to 1, the random walk mainly occurs according to the transfer matrix *M*. When *d* is close to 0, the random walk is dominated by random probability. Each component of *R* is the PR value of each node, which is given by:

5 $$\begin{array}{*{20}c} {PR\left( {v_{i} } \right) = d\left( {\mathop \sum \limits_{{v_{j} \in M\left( {v_{i} } \right)}} \frac{{PR\left( {v_{j} } \right)}}{{L\left( {v_{j} } \right)}}} \right) + \frac{1 - d}{n},\;i = 1,2, \ldots ,n,} \\ \end{array}$$

where ${v}_{i}$ and ${v}_{j}$ represent node i and node j, $M\left({v}_{i}\right)$ denotes the set of nodes pointing to ${v}_{i}$ and $L\left({v}_{j}\right)$ denotes the outdegree of node ${v}_{j}$. Table 1 summarizes the commonly used parameters in this paper.

**Table 1:** Commonly used parameters.

Notation	Explanation
*n*	Number of network nodes
*M* ∈ ℝ^*n*×*n*^	State transition matrix of network nodes
*G*	Community
*R*^*n*×*1*^	Stationary distribution of the general random walk Markov chain
*A*	Directed graph
*V*	Vertices of cyberspace
*E*	Directed edges of cyberspace
**1**	Column vector of size n in which all elements are equal to 1

### Quality metrics for cyberspace communities

In this study, modularity (Q)^43^ and normalized mutual information (NMI)^44^ are used as metrics for community structure. Q is a metric that characterizes the degree of connection within a community, i.e., the strength of connectivity among nodes in the community. Based on a large body of experimental evidence, it has been determined that Q > 0.3 is indicative of a strong community structure^45,46^. NMI is a metric used to evaluate the similarity of the calculated clustering solution to the actual community structure, as it measures the clustering similarity of two clustering solutions.

### The community partitioning algorithm for cyberspace mapping

In this study, a community partitioning algorithm for cyberspace mapping, known as PR-LFM, was constructed by combining an improved LFM algorithm with PR. This algorithm has three distinct stages: seed node selection, local community expansion, and the partitioning of overlapping nodes. The procedures of the algorithm are as follows:

- Step 1. Seed node selection

In the original LFM algorithm, seed nodes are randomly selected, which results in unstable partitioning results. Given that nodes with high degree centrality are usually key nodes that are important for the dissemination of information, the seed nodes were selected based on the degree centrality. The degree centralities of all nodes in the community are calculated using Eq. (6), and the nodes are then ranked accordingly. The node that has the highest degree centrality is selected as the seed node. This ensures that the same seed nodes are chosen in each run, which assures stable results.

6 $$\begin{array}{c}{DC}_{i}=\frac{{k}_{i}}{n-1}\end{array}$$

- Step 2. Local community expansion

The node with the highest degree centrality from Step (1) is selected as the seed node to start community expansion. The fitness of this community is then calculated using the following equation:

7 $$\begin{array}{c}{f}_{G}=\frac{{k}_{in}^{G}}{{\left({k}_{in}^{G}+{k}_{out}^{G}\right)}^{\alpha }},\end{array}$$

where ${k}_{in}^{G}$(${k}_{out}^{G}$) is the sum of the weights of the edges inside (outside) community *G*, and *α* is the resolution parameter that controls the size of the community. The hierarchy of the community structure can be analyzed by selecting different values of *α* to partition the network (When the *α* value is low, fewer communities are divided. When the *α* value is large, more communities are divided).

For any node in the network, a fitness function ${f}_{G}^{i}$ may be defined for the fitness of node *i* for the community *G*:

8 $$\begin{array}{c}{f}_{G}^{i}={f}_{G+i}-{f}_{G-i},\end{array}$$

where ${f}_{G-i}$ and ${f}_{G+i}$ are the fitnesses of community *G* before and after node *i* is added, respectively. The value of ${f}_{G}^{i}$ can be used to determine whether the node joins the community $G$ or not. If ${f}_{G}^{i}>0$, the addition of node $i$ increases the fitness of community *G*; hence, the node should be retained in community *G*; otherwise, the node should be removed from community *G*. The expansion of community *G* terminates after all nodes have been traversed^25^. The node with the highest degree of centrality among the unpartitioned nodes is then selected as a seed node for expansion into a new community. This process is repeated until all nodes have been partitioned into at least one community.

- Step 3. Partitioning of overlapping nodes

**Figure 3:**
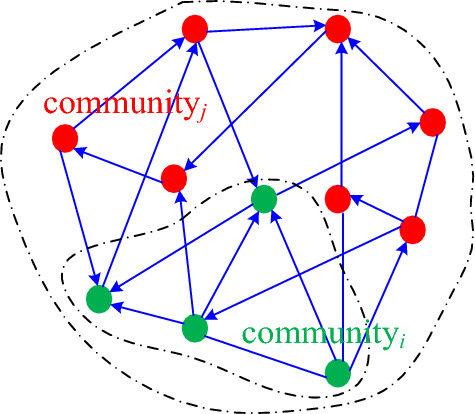
Fully overlapping communities. Nodes in community_i_ exist entirely in community_j_.

Based on the communities that were detected in Step (2), if there exists some community_*i*_ that is entirely a subset of another community_*j*_ (green and red nodes in Fig. 3, respectively), it is necessary to traverse all communities to eliminate all instances of community_*i*_.

**Figure 4:**
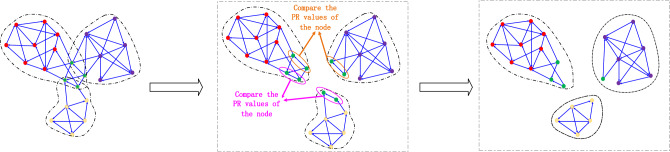
Community detection model for cyberspace mapping. The green overlapping nodes are partitioned into the red, purple, and yellow communities to form a new community structure. Then, the PR value of each overlapping node in each community is calculated, and the node is ultimately retained in the community with the highest PR value.

Subsequently, the communities that contain overlapping nodes are identified from the remaining communities, and the overlapping nodes are then partitioned into their rightful communities. To this end, the PR value of each overlapping node in each community is calculated, and the node is ultimately retained in the community with the highest PR value. An example is shown in Fig. 4.

**Figure 5:**
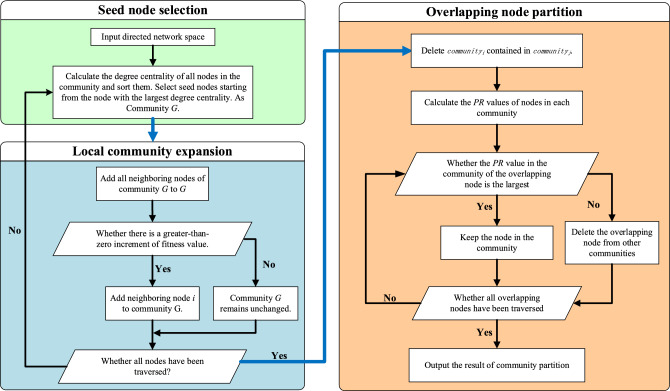
Algorithmic flowchart for the PR-LFM community detection algorithm. The flow chart describes our algorithm in detail, all nodes go through three steps, and the final output node is the partitioned results.

Steps (2) and (3) are repeated until all nodes have been partitioned into a community. A flowchart of the algorithm is shown in Fig. 5. In this study, NetworkX,^47^ Gephi^48^, and Echarts network analysis packages are used to implement the community detection algorithm.

## Experiments and analysis

This study compares the PR-LFM algorithm with the traditional community partitioning algorithm to verify the reliability of the proposed algorithm in small-scale network partitioning. Subsequently, it performs community partitioning based on the real cyberspace router topology data. Experiments demonstrate that the proposed algorithm can retain the real structure of the community better when partitioning the data of large-scale network resources, which can help us better explore the internal connections.

### Validation of the PR-LFM algorithm’s reliability

To verify the reliability of the PR-LFM algorithm and ascertain its practical value, the *Q*, NMI, and time of the PR-LFM algorithm are compared with those of other non-overlapping community detection algorithms, such as the GN, Louvain, Infomap, and LPA algorithms. These community detection tests were performed on the classic Karate^49^, Dolphins^50^, Lesmis^51^, Polbooks^52^, and small scale real router networks. The details of these classic networks are presented in Table 2, and the router network information is shown in Table 3.

**Table 2:** Classic networks.

Network name	Nodes	Edges	Community	Average network degree	Map density
Karate	34	78	2	4.588	0.139
Dolphins	62	159	2	3.968	0.084
Lesmis	77	254	–	2.156	0.029
Polbooks	105	441	3	2.752	0.026

**Table 3:** Features of router networks.

	Network I	Network II	Network III	Network IV	Network V	Network VI	Network VII	Network VIII
Nodes	100	143	175	195	200	226	300	350
Edges	288	222	277	285	620	359	906	978

**Figure 6:**
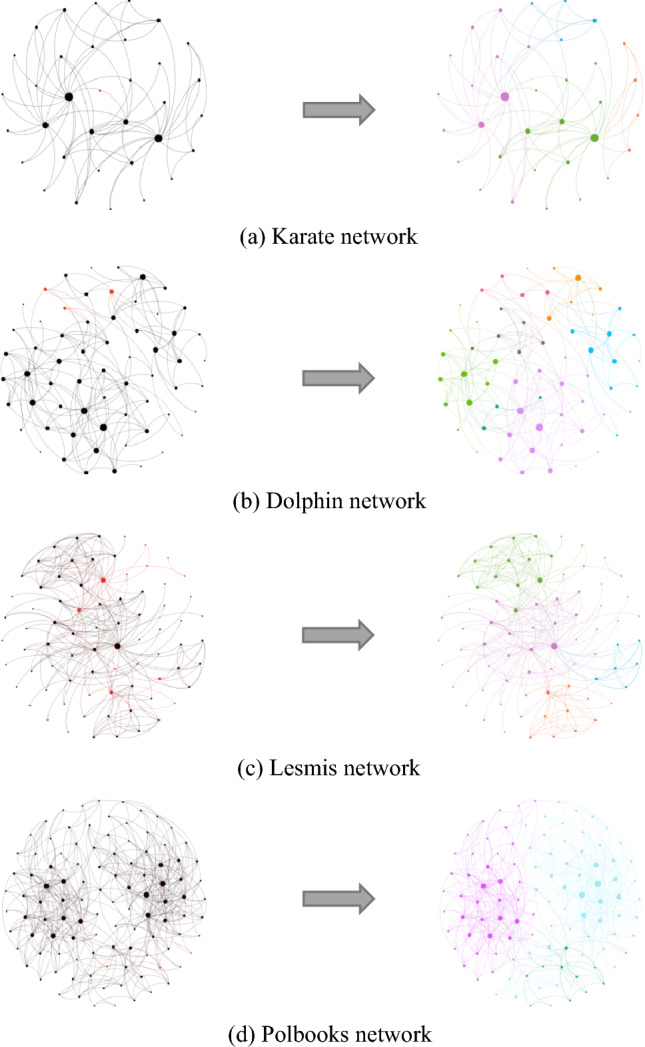
Results of community detection by the PR-LFM algorithm on classic networks. In the figures on the left, the overlapping nodes are colored red. The results of community division are shown on the right, where all nodes in the same community have the same color. The sizes of the nodes are proportional to their degree centrality.

The Karate network comprises members of a karate club^49^. Our proposed algorithm partitioned this network into four communities and found one overlapping node. The Dolphin network, constructed by Lusseau et al. in New Zealand, models the habits of bottle-nosed dolphins^50^. Our algorithm partitioned this network into seven communities, identifying four overlapping nodes. The Lesmis network represents interactions among characters in Victor Hugo's *"Les Misérables."*^51^ This network was partitioned into four communities, with 12 overlapping nodes. The Polbooks network was constructed by analyzing the political leanings of purchasers of American political books in the Amazon online bookstore^52^. This network was partitioned into three communities, with three overlapping nodes. Finally, the overlapping nodes were retained in the community for which they had the highest *PR* value, as shown in Fig. 6.

To evaluate the reliability of the algorithm, the *Q* and NMI of the PR-LFM algorithm on said classic networks were compared to those of other non-overlapping community detection algorithms, as shown in Table 4. It can be observed that the PR-LFM algorithm produces results that are comparable with those of conventional non-overlapping community partitioning algorithms in the aforementioned classic networks. In terms of *Q*, the PR-LFM algorithm is the best performer on the Karate network, with *Q* = 0.4156. Furthermore, the *Q* values of the PR-LFM algorithm for the Dolphin network and the Polbooks network are higher than those of the Infomap and LPA algorithms. In terms of NMI, the PR-LFM algorithm is the best performer on the Polbooks network, with NMI = 0.55657. Moreover, the NMI of the PR-LFM algorithm on other networks is comparable to those of the conventional algorithms.

**Table 4:** NMI and Q values of the PR-LFM algorithm and conventional community detection algorithms on classic networks.

		GN	Louvain	Infomap	LPA	PR-LFM
Karate	Q	0.4013	0.41511	0.35051	0.35470	**0.41560**
	NMI	0.57983	0.70714	0.56444	**0.72096**	0.60214
Dolphin	Q	0.51938	**0.51958**	0.39977	0.45633	0.49830
	NMI	**0.55416**	0.47425	0.45441	0.46122	0.44658
Lesmis	Q	0.53807	**0.55827**	0.47092	0.52668	0.51171
	NMI	–	–	–	–	–
Polbooks	Q	0.5168	**0.52639**	0.39786	0.48114	0.49927
	NMI	0.55845	0.55609	0.46804	0.53410	**0.55657**

Significant values are in [bold].

**Figure 7:**
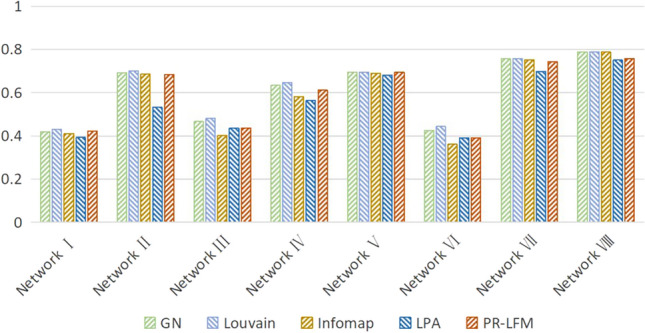
Comparison results of PR-LFM and comparison algorithm Q on the router network.

Since there is no real community partition result in the router network, the reliability of the proposed algorithm is analyzed in terms of modularity and running time. A comparison of the PR-LFM algorithm proposed in this study and the traditional non-overlapping community, showing the module degree Q and running time of the algorithm during community partitioning on the router network, is presented in Table 5 and Fig. 7.

**Table 5:** Comparison of the running time of router networks on different community partitioning algorithms (seconds).

	GN	Louvain	Infomap	LPA	PR-LFM
Network I	5.64736	0.01079	0.01248	0.00996	0.01296
Network II	4.55494	0.01496	0.01358	0.01331	0.01392
Network III	15.36345	0.02172	0.01747	0.01894	0.02393
Network IV	12.73726	0.02389	0.02247	0.02046	0.03293
Network V	28.70579	0.02696	0.02745	0.02496	0.03438
Network VI	47.24140	0.02798	0.02897	0.02647	0.03472
Network VII	92.06524	0.04775	0.04893	0.03494	0.05120
Network VIII	128.62424	0.04985	0.05192	0.04742	0.05996

As depicted in Fig. 7, when the proposed algorithm divides communities for real router networks, the modularity value consistently surpasses 0.3, indicating that when the proposed algorithm divides communities for router resources in cyberspace, it takes into account the hierarchical and overlapping characteristics of communities in cyberspace and the directivity of transferring resource information, the community structure in cyberspace can still be well preserved. By comparing the modularity with other traditional community partitioning algorithms, we can see that the proposed algorithm can achieve the effect of traditional community partitioning algorithms. Simultaneously, Table 5 reveals that the operation efficiency of the proposed algorithm is significantly higher than that of the GN algorithm. The running time is basically consistent with that of other traditional algorithms, indicating that the algorithm can still be applied to large-scale networks after comprehensive consideration of the characteristics of resource data in the cyberspace.

The experimental results demonstrate that the PR-LFM can achieve the partition effect of the traditional non-overlapping algorithm when classifying resources in the cyberspace. By considering the overlap between communities in real cyberspace, the algorithm proposed in this study can control the number of communities through the parameter α value of community size and consider the direction of resource information transmission in cyberspace through the PageRank algorithm. Compared with the traditional community division algorithm, the algorithm proposed in this study effectively retains the features in the cyberspace. Simultaneously, it achieves a level of efficiency comparable to mainstream algorithm in community division and can be applied to large-scale cyberspace resource data, demonstrating its research value.

### Application of the PR-LFM algorithm to cyberspace mapping

**Figure 8:**
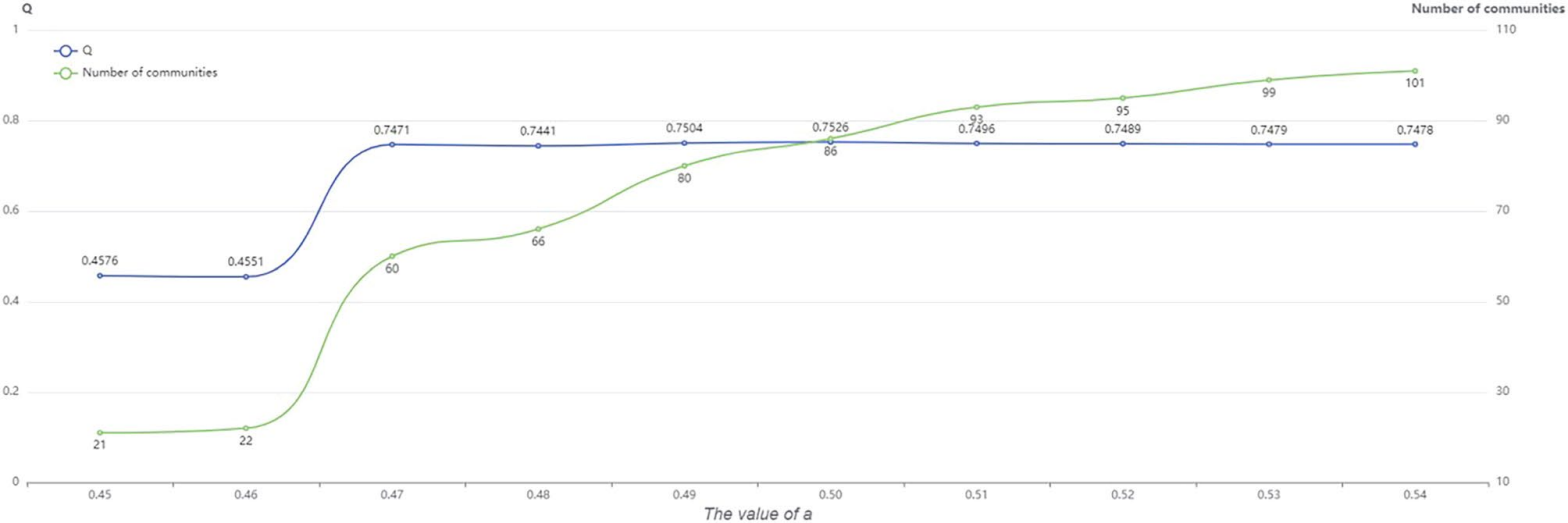
Results of community detection on a real router network using the PR-LFM algorithm with different values of α.

Following the effectiveness of the PR-LFM algorithm was validated, a community partitioning test was performed using real router topology data. This dataset contains 4677 router nodes and 6123 edges comprised of child and parent nodes. The experimental procedures are as follows:

1. Following multiple trials, it was found that community partitioning can be performed effectively using *α* values of [0.45–0.54]. Furthermore, the number of detected communities depended on* α*, which reflects on the hierarchical nature of the community structure. To optimize the algorithm, *Q* was plotted against *α*, as shown in the graph of Fig. 8.

Figure 8 illustrates that *Q* always exceeds 0.3 when *α* = [0.45, 0.54], indicating that the algorithm produces good results for these values of *α*. As *Q* increases significantly when *α* increases from 0.46 to 0.47, it is implied that *α* = 0.47 gives the ideal degree of node connectivity and separation within the detected communities. From *α* = 0.47 to *α* = 0.54, *Q* only fluctuates slightly around 0.75, which shows that the intra- and inter-community structure was already stable at *α* = 0.47. *Q* reached its maximum (0.7526) when *α* was 0.50.

**Figure 9:**
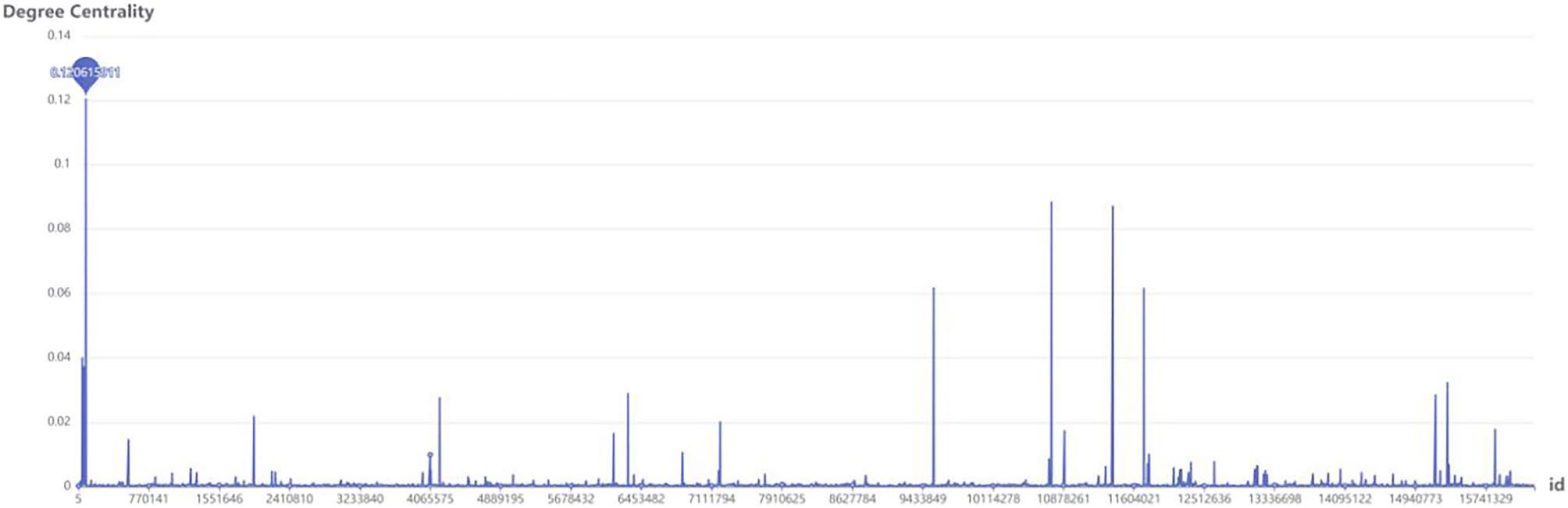
Degree centrality of different router nodes.

(2) The degree centralities of all router nodes that correspond to *α* = 0.50 were calculated using Eq. (6), as shown in Fig. 9. Here, ID 1980 has the highest degree centrality, at 0.120615911. Therefore, this node was used as the initial seed node for local community expansion.

The router network was subsequently partitioned based on fitness values, which yielded 86 communities. 80 had overlapping router nodes, and 1359 router nodes, or approximately 29.06% of all router nodes, were present in two or more communities.

**Figure 10:**
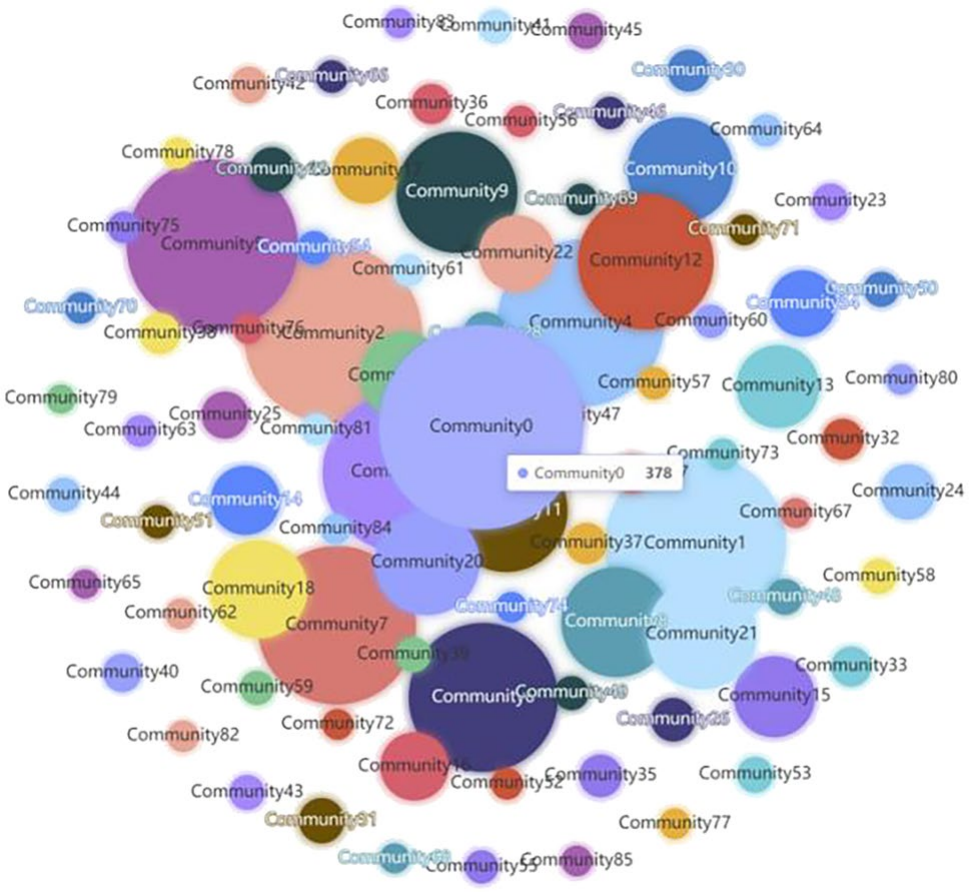
Results of community detection on a real router network using the PR-LFM algorithm. Each circle represents a different community, and the size of the circle represents the number of nodes in the community. For instance, Community_0_, which has the most router nodes (378), presents the largest circle.

(3) The overlapping router nodes were assigned to their respective communities by comparing their PR values for each community and assigning the node to the community in which their PR was higher. This process was repeated until each node belonged to just one community. The PR-LFM algorithm ultimately partitioned the 4677 router nodes into 86 communities, as shown in Fig. 10.

To test the reliability of PR-LFM on large-scale networks, the PR-LFM algorithm was compared to the aforementioned non-overlapping algorithms in terms of *Q* (except for the GN algorithm, as it has a time complexity of O(n^3^) where n is the number of nodes, making it unsuitable for large-scale networks). Table 6 indicates that the PR-LFM algorithm is second only to the Louvain algorithm in terms of *Q*. Unlike other algorithms, the PR-LFM algorithm can revisit a node multiple times during community partitioning and directly evaluate each node’s contribution to its community, thus enabling the detection of node overlaps. Moreover, the assignment of overlapping nodes to the communities in which they are the most important helps preserve the community structure.

**Table 6:** Q of the PR-LFM algorithm and conventional community detection algorithms on an actual router network.

Algorithm	*Q*
GN	–
Louvain	**0.7758**
Infomap	0.6862
LPA	0.6553
PR-LFM	0.7526

Significant values are in [bold].

## Conclusion

To account for the hierarchical and overlapping nature of the community structure of cyberspace and the directionality of its information transfers, we propose the PR-LFM algorithm, a community partitioning method that combines an improved LFM algorithm with the PR algorithm. To account for community overlaps in actual cyberspace, the seed nodes are determined using degree centrality, and the nodes are partitioned into overlapping communities based on a fitness function. To account for the directional nature of information transfer between router nodes, the PR algorithm is used to calculate PR values for overlapping nodes in each of their communities, which are retained in the community where they are the most important (i.e., have the highest PR value). Thus, the overlapping cyberspace communities are partitioned into non-overlapping communities. Experiments were conducted on the classic Karate, Dolphins, Lesmis, and Polbooks networks and real router topology data, which led to the following conclusions:

1. Based on comparisons in terms of *Q*, NMI, and time, the PR-LFM algorithm was found to match the performance of conventional non-overlapping community partitioning algorithms. Furthermore, the PR-LFM algorithm produces stable community partitioning results, as it uses degree centrality to select seed nodes. Therefore, the PR-LFM algorithm is reliable for the partitioning of cyberspace router nodes.
2. The PR-LFM algorithm can account for community overlaps during the community partitioning process. When partitioning 4677 router nodes, it detected 1359 overlapping nodes, representing 29.06% of all router nodes. Unlike conventional non-overlapping community detection algorithms, PR-LFM would subsequently partition these overlapping nodes into distinct communities; this helps preserve the community structure.
3. The PR-LFM algorithm accounts for the directionality of information transfers between routers in cyberspace by using the PR algorithm to evaluate the importance of each node in the network, which allows the algorithm to accurately characterize router node interactions.
4. As the non-overlapping communities derived from this algorithm account for the hierarchy, overlap, and directionality of community structures, it is suitable for mapping large-scale cyber-networks and has wide-ranging applications in social network analysis, bioinformatics, recommendation systems, and natural language processing.

The goal of this study is to divide the resource node community in cyberspace to manage, maintain, and optimize the mapping data of cyberspace resources. The results demonstrate that the proposed algorithm considers the hierarchy, overlap, and direction of information transmission in the real network structure and can retain the structure in the real cyberspace. Considering that several network resource data have the attributes of geographic space, future research can explore the attributes of network, and geographic space can be combined as the basis for community division. This approach would enable community structure division to consider the characteristics of both network and geographic space, as well as promote the cross-domain application of data and resource information mining. Simultaneously, due to the consideration of community structure characteristics in cyberspace, the proposed algorithm exhibits improved time complexity when compared to the LFM algorithm. Therefore, the following work can focus on optimizing the algorithm and reducing the time complexity to enable efficient handling of large-scale cyberspace resource data.

## Data Availability

The data used in this study were all derived from citable primary sources, and the actual datasets used in this study may be obtained from the corresponding author.
